# Sebaceous nevus of Jadassohn: review and clinical-surgical approach^☆^

**DOI:** 10.1016/j.abd.2021.11.001

**Published:** 2022-07-19

**Authors:** Manoel Pereira da Silva Neto, Barbara Rodovalho de Assis, Gustavo Rodrigues Andrade

**Affiliations:** a Plastic Surgery Department, Universidade Federal do Triângulo Mineiro, Uberaba, MG, Brazil; b Universidade Federal do Triângulo Mineiro, Uberaba, MG, Brazil

**Keywords:** Hamartoma, Mosaicism, Neurocutaneous syndromes, Nevus, sebaceous of Jadassohn, Skin

## Abstract

**Background:**

Nevus sebaceous of Jadassohn is defined as a rare congenital malformation characterized as a non-hereditary hamartoma of the adnexal structures of the skin. Its etiology is not yet well understood, but it is believed to be related to post-zygotic mutations in the *HRAS*, *NRAS* and *KRAS* genes.

**Objective:**

To describe the clinical manifestation of nevus sebaceous, as well as the main management techniques addressed in the medical literature. Moreover, the present study discusses a case report of a congenital linear nevus in the left retroauricular region found in a male patient, without extracutaneous manifestations.

**Method:**

A narrative review of the literature was carried out.

**Discussion:**

Nevus sebaceous occurs as lesions with a linear or oval appearance, with a smooth or verrucous texture, generally alopecic and with very variable color. Moreover, nevus sebaceous is one of the components of the so-called linear nevus syndrome or Schimmelpenning-Feuerstein-Mims syndrome, which is associated with multisystemic complications. The treatment of the lesions is still controversial; however, most experts indicate surgical excision as the most frequently adopted treatment method, in addition to multidisciplinary follow-up when the diagnosis of Schimmelpenning-Feuerstein-Mims syndrome is established.

**Conclusion:**

The linear nevus syndrome constitutes a rare manifestation; however, its diagnosis should be considered in children born with nevus sebaceous. There is no consensus yet on the best therapy, but surgical removal has shown to be a viable option.

## Introduction

Nevus sebaceous, also known as organoid nevus, Jadassohn nevus, or pilosyringosebaceous nevus, is characterized as a rare, non-hereditary, congenital hamartoma, resulting from hyperplasia of epithelial, sebaceous, follicular, and apocrine elements of the skin.^1^, ^2^, ^3^, ^4^ The etiology of this disease has not yet been fully clarified and, therefore, needs further investigation. However, recent studies suggest the association of a post-zygotic somatic mutation related to the *HRAS* (chromosome 11p15), *NRAS* (chromosome 1p13) and *KRAS* (chromosome 12p12) genes^5^, ^6^ in the genesis of this condition, as they condition the cell proliferation process.^6^, ^7^ The clinical manifestation occurs as plaques with partial or complete alopecia, with a linear or oval shape, and color ranging from skin-colored, to yellowish-orange or brownish-black, with a smooth, nipple-like or verrucous appearance, depending on the degree of lesion development.^3^, ^8^, ^9^ The commonly affected regions include the scalp, followed by the preauricular area, face, and cervical regions. However, several studies have reported its occurrence in other less frequent areas, such as mucosa, trunk and extremities, so that, when found in these sites, the lesions are distributed following the orientation of the Blaschko lines.^2^, ^3^, ^10^ Nevus sebaceous may be related to extracutaneous manifestations affecting different organs and, in this case, it constitutes a more complex clinical picture, being called linear nevus syndrome or Schimmelpenning-Feuerstein-Mims syndrome.^3^, ^6^

## Objective

The present study aims to report the main aspects of nevus sebaceous and nevus sebaceous of Jadassohn syndrome, outlining their characteristics, clinical manifestations and emphasizing therapeutic alternatives.

## Method

This is a literature review study, with a narrative focus. A search was carried out on the PubMed database in May 2021, restricting the results to the last five years (2016‒2021), with the following expressions in English: “Nevus sebaceous”, “sebaceous nevus”, “syndrome Schimmelpenning” and “sebaceous nevus Jadassohn”.

The following inclusion criterion was employed: articles strictly related to the topic. The screening of articles was based on titles and/or abstracts, availability of the full article and publications in English, Portuguese, Spanish and French. The articles that did not meet the previously established criteria were removed. A total of 128 articles were found and, of these, 64 were selected and 64 were excluded. Of the 64 selected articles, after reading and critical analysis, 24 were chosen to be included in the present study. Subsequently, in order to broaden the study perspective, the works by Happle,^11^ Basu et al.,^12^ McCalmont^1^ and Kang et al.^13^ were also included.

## Historical background

In 1895, the German dermatologist Josef Jadassohn described the organoid nevus, which is a subclassification of the epidermal nevus, as a congenital malformation involving adnexal structures, mainly the sebaceous glands.^3^, ^14^, ^15^, ^16^ Subsequently, in 1957, Gustav Schimmelpenning performed the evaluation of a patient with skin lesions and neurological impairment manifestations caused by cranial malformation and, due to the fact that this condition did not correspond to any other previously described clinical picture, Schimmelpenning categorized it as a neurocutaneous phakomatosis.^5^, ^11^ In 1962 Feuerstein and Mims reported a case of a linear nevus associated with seizures and intellectual disability symptoms.^5^ Since then, the so-called classic triad used for the diagnosis has been created, which consists of neurological impairment, seizures, and intellectual disability, associated with the presence of a nevus sebaceus.^11^ However, subsequent studies have shown that the extracutaneous manifestations of the Schimmelpenning-Feuerstein-Mims syndrome are much more diverse.^3^

## Case report

**Figure 1:**
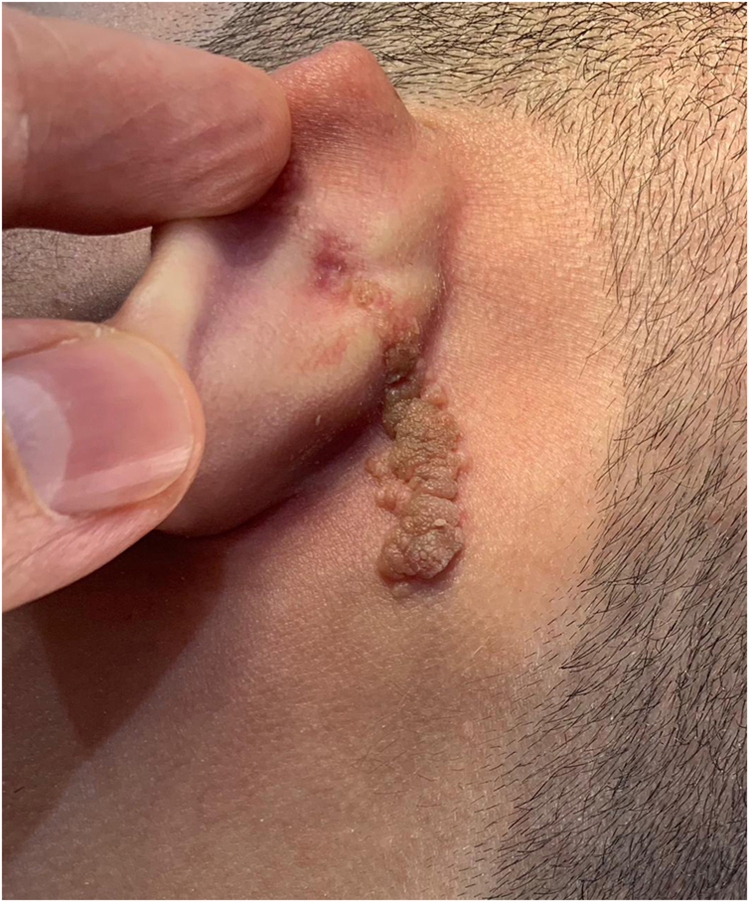
Linear lesion with a slightly reddish and brownish color and a verrucous appearance.

**Figure 2:**
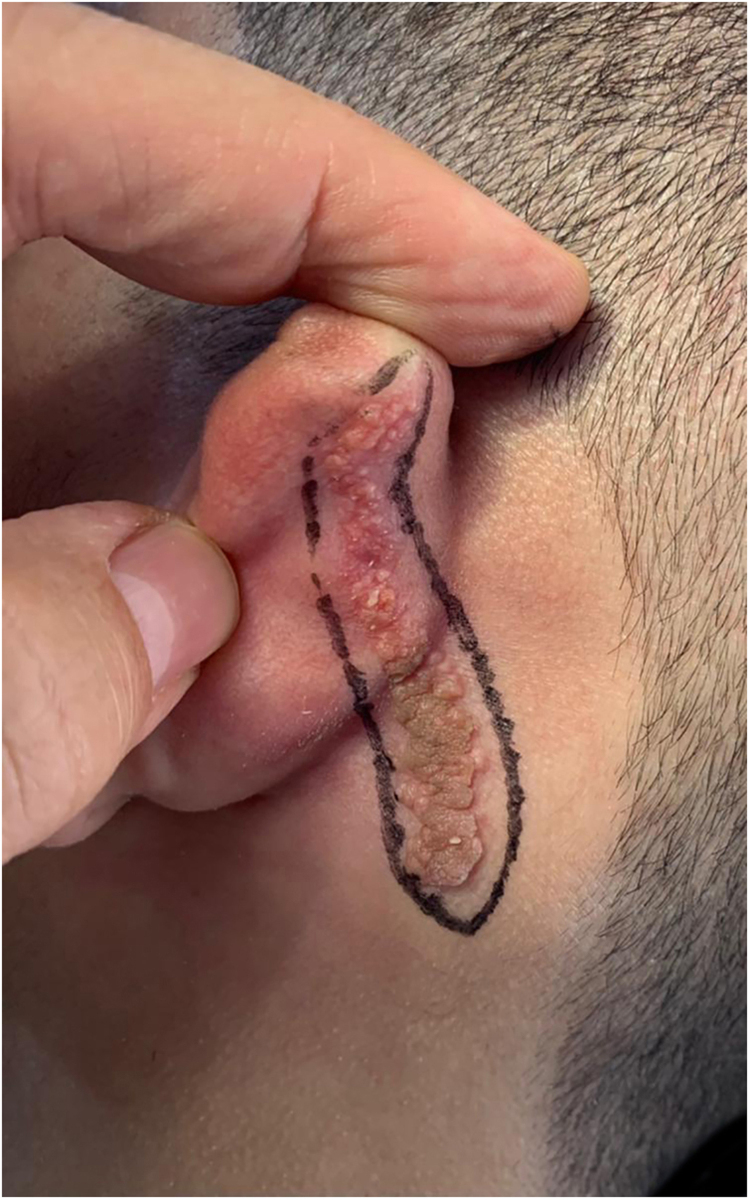
Pre-surgical marking.

**Figure 3:**
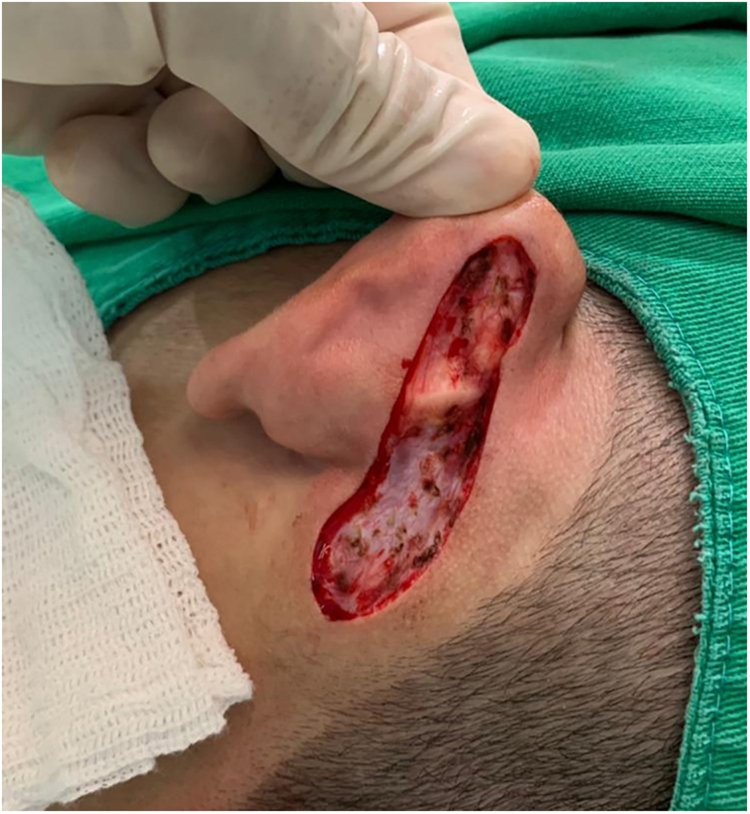
Lesion excision.

**Figure 4:**
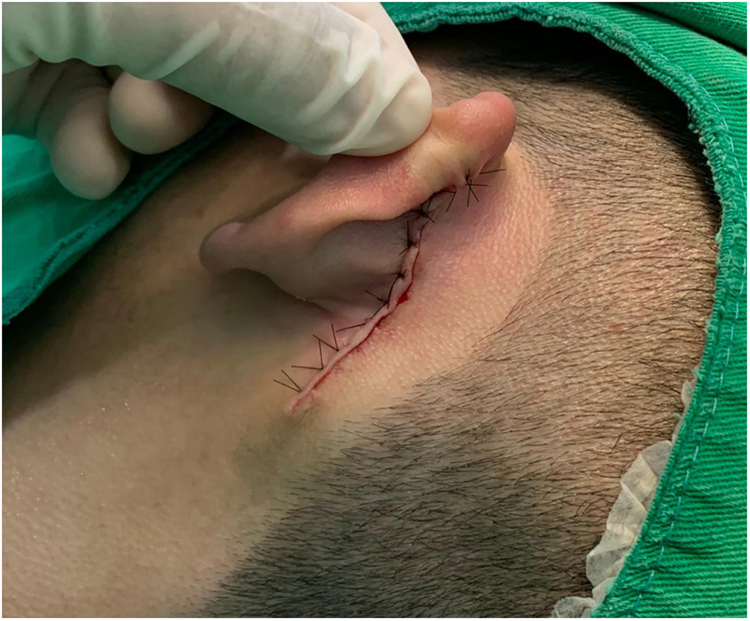
Primary closure.

An 18-year-old male patient, healthy and with no other complaints, was evaluated for a unilateral congenital lesion on the left retroauricular region. Physical examination revealed a linear lesion consisting of pigmented papules, slightly reddish and brownish in color, with a verrucous appearance and well-defined borders (Fig. 1). Clinical evaluation disclosed no lesion progression, except for the patient's own physical growth. Systemic evaluation revealed no abnormalities. The patient wanted the lesion removed for aesthetic reasons and because of repeated trauma. Moreover, there was a parental concern due to the positive history of the death of a family member due to melanoma, who also had nevi. The lesion was surgically excised in an outpatient setting, with a safety margin of 5 mm up to the level of the muscular fascia (Figure 2, Figure 3), with primary closure (Fig. 4).

**Figure 5:**
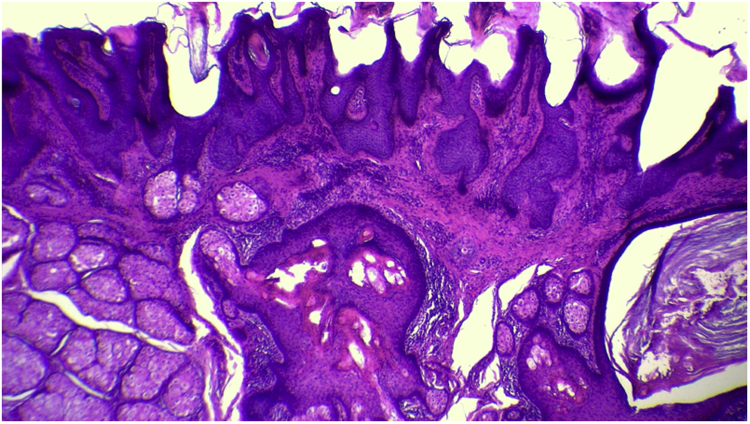
Panoramic histopathological view showing mild hyperkeratosis, accompanied by acanthosis and papillomatosis (Hematoxylin & eosin, ×40).

**Figure 6:**
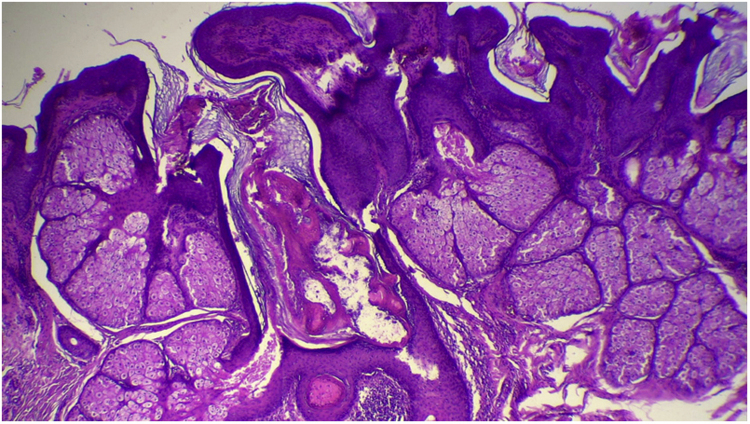
Hyperplasia of sebaceous glands localized close to the epidermis (Hematoxylin & eosin, ×40).

**Figure 7:**
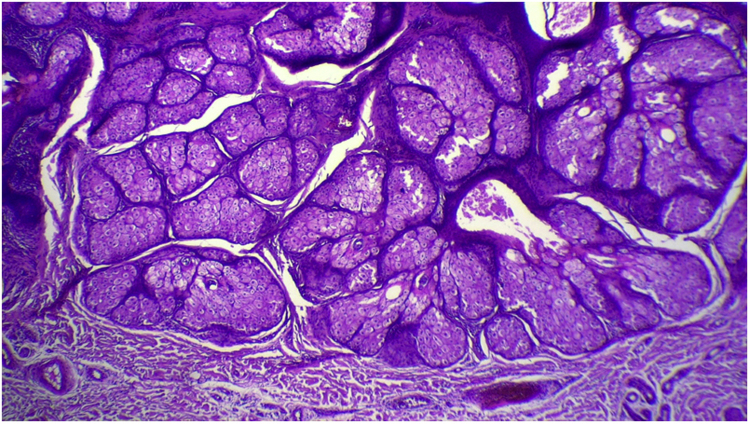
Detail of sebaceous glands hyperplasia (Hematoxylin & eosin, ×40).

The anatomopathological evaluation disclosed mild hyperkeratosis, accompanied by acanthosis due to the increased thickness of the epidermis. Moreover, there was papillomatosis demonstrated by intense sinuosity of the dermo-epidermal junction and clinically corroborated by the verrucous aspect of the lesion (Fig. 5). Hyperplasia of the sebaceous glands was also observed, with these adnexal structures localized more closely to the surface of the skin (Figure 6, Figure 7). Therefore, the histopathological findings were consistent with nevus sebaceus.

## Epidemiology

The incidence of nevus sebaceus in newborns is estimated at 0.1% to 0.3%,^13^ with no predilection for sex or ethnicity.^2^, ^12^ Although familial cases have been described, the lesion manifests randomly.^13^ Regarding the main areas of involvement, 62.5% of the lesions are found on the scalp, 24.5% on the cephalic segment, 4.7% on the trunk, and 4.2% on the cervical region.^17^ In addition to aesthetic impairment, one of the major concerns related to this disease is associated related to its capacity to generate secondary neoplasms.^18^ Studies indicate that this phenomenon occurs in approximately 10% to 20% of the cases, mostly in patients over 40 years of age.^12^, ^19^ However, most secondary neoplasms are benign, so only approximately 3% of cases have some degree of malignancy and are considered rare incidences.^19^ The most frequent malignant tumors include basal cell carcinoma (1.1%) and squamous cell carcinoma (0.57%), followed by sebaceous carcinoma and apocrine carcinoma.^20^ Secondary neoplasms with lower incidence rates reported in the literature include sebaceous carcinoma, squamous cell carcinoma, microcystic carcinoma, and melanoma.^2^, ^15^, ^16^, ^19^^,^^21^

## Clinical presentation

Nevus sebaceous is present since birth or shortly thereafter.^2^, ^16^ The clinical manifestation of the lesions can be subdivided into three stages or phases of clinical evolution based on the morphological appearance and age group of the affected individual, even though age does not have a definite relationship with the clinical manifestations.^21^ The first stage corresponds to the disease manifestation after birth and extends throughout childhood, a period in which it appears as a disc-shaped lesion or small associated spots, smooth or partially hairless, ranging in color from pale skin color, slightly yellowish, yellowish-pink, yellowish-orange, or brown.^6^, ^15^ During puberty, the second stage is characterized by hyperplasia of the sebaceous glands and other adnexal structures due to hormonal influence.^6^, ^15^ Studies have indicated the presence of androgen receptors in nevus sebaceous,^4^ so that lesions take on a verrucous appearance and a more intense color, due to the processes of hyperkeratosis, papillomatosis, and acanthosis.^6^, ^14^, ^15^ Finally, the third stage is seen in the adulthood, when there is a higher risk of secondary neoplasm development.^6^, ^15^

A linear nevus is usually reported in Schimmelpenning-Feuerstein-Mims syndrome and, as a component of multisystemic disorders, which have been previously described in a wide variety of organs, such as the nervous, ocular, cardiovascular, muscular, urogenital systems, and bones, among others.^14^, ^15^, ^22^, ^23^ Aiming to facilitate the identification and clinical diagnosis, Table 1 shows the main disorders that have been previously identified and related to Schimmelpenning-Feuerstein-Mims syndrome, among which the most frequently reported in the literature are: hypophosphatemic rickets, intellectual disability, and cognitive impairment, coloboma and strabismus.^5^, ^11^, ^22^

**Table 1:** Extracutaneous clinical manifestations of the nevus sebaceous syndrome.^5^, ^11^, ^22^

**Skeletal**	Kyphoscoliosis
	Craniofacial defects
	Limb deformities
	Hip dislocation
	Frontal bulge
	Hypophosphatemic rickets
**Neurological**	Agenesis of the corpus callosum
	Agyria, microgyria or cortical pachygyria
	Generalized brain atrophy
	Seizures
	Intellectual disability
	Cognitive impairment
	Cerebral vessel dysplasia
	Pineal hamartoma
	Left thalamic hamartoma
	Hemimegaloencephaly
	Cerebral heterotopia
	Dandy-Walker syndrome
**Ocular**	Coloboma
	Optic nerve defects
	Strabismus
	Epibulbar lipodermoid
	Microphthalmia
	Corneal opacity
**Genitourinary tract**	Cryptorchidism
	Hydronephrosis
	Hypospadias
	Nephroblastomatosis
	Ureteropelvic junction obstruction
	Cystic kidney
	Horseshoe kidney
	Duplicated renal collecting system
	Testicular and paratesticular tumors
**Cardiovascular**	Coarctation of the aorta
	Ventricular septal defect
	Aortic hypoplasia
**Intraoral**	Tooth aplasia
	Bone cysts
	Hypoplastic enamel
	Hemihyperplasia of the tongue
**Lymphatic system**	Lymphedema
	Chylothorax

### Histopathological findings

Regarding the pathological findings, the main assessment to guide the therapeutic approach is related to the analysis of the risk of developing secondary neoplasms, which is low for secondary carcinomas and high for benign neoplasms.^2^ In this regard, the main reported benign neoplasms are trichoblastoma (TB) and syringocystadenoma papilliferum (SCAP), followed by trichilemmoma, sebaceous adenoma, desmoplastic trichilemmoma, apocrine adenoma, and poroma.^2^, ^18^, ^23^

Trichoblastoma is defined as a trichogenic tumor, formed by the proliferation of follicular germ cells.^16^ On histological examination, trichoblastoma shows small cells of round to oval morphology, grouped and separated by a fibrous stroma, in a stratified squamous epithelium and areas of necrosis with calcification.^24^

Syringocystadenoma papilliferum is a neoplasia of the apocrine and eccrine sweat glands and manifests as isolated patches or as multiple nodules containing vesicles or exudate.^16^ Histopathologically, the SCAP is characterized as an apocrine adnexal tumor with papillary projections and cystic ducts, which are covered by columnar or cuboidal cells with basophilic cytoplasm, often having a connection with the epidermis.^12^

As for malignant neoplasms, basal cell carcinoma (BCC) is the most frequently reported, although its occurrence is considered rare.^2^ BCC has significant histological similarities to trichoblastoma so assumptions indicate an overestimation of its actual incidence as a secondary neoplasm.^2^ The differentiation relies on the presence of a myxoid stroma in addition to retraction of the stroma around the basaloid cell clusters, findings typical of basal cell carcinoma.^24^

Regarding the genetic perspective, several studies have indicated a relationship between the appearance of nevus sebaceous, alone or as a multisystemic syndrome, and mutations in the *HRAS*, *KRAS,* and *NRAS* genes, so that the amino acid glycine is replaced by the amino acid arginine, which in turn leads to activation of the MAPK and PI3K-AKT pathways,^7^, ^16^ with a consequent increase in cell proliferation in mutated cells,^6^, ^7^ which was confirmed through the genetic analysis of blood cells and tissues from unaffected areas, corroborating the theory of genetic mosaicism.^15^, ^25^ Table 2 below depicts the most recurrent neoplasms in medical literature associated with nevus sebaceous.^2^, ^12^, ^23^

**Table 2:** Main secondary neoplasms.^2^, ^12^, ^23^

Benign	Malignant
Sebaceous adenoma	Adnexal carcinoma
Seborrheic keratosis	Apocrine carcinoma
Pigmented eccrine poroma	Basal cell carcinoma
Hidradenoma	Squamous cell carcinoma
Infundibuloma	Mucoepidermoid carcinoma
Osteoma	Sebaceous carcinoma
Proliferation of basaloid cells	Keratoacanthoma
Sebaceoma (Sebaceous epithelioma)	
Syringocystadenoma papilliferum	
Trichoblastoma	
Desmoplastic trichilemmoma	
Verruca vulgaris	

## Treatment

As with other epidermal nevi, nevus sebaceous can be permanently treated with full-thickness excision^13^ in patients who complain of aesthetic and psychological discomfort.^15^ Lesion removal for prophylactic purposes is still widely debated.^13^ Seeking to highlight and evaluate this discussion, the article by Wali, Felton, and McPherson, published in 2018, addresses research carried out through a questionnaire sent to dermatologists and plastic surgeons in the United Kingdom aiming to determine the best current intervention for the management of nevus sebaceous. ^26^ However, the results differed between the two groups of specialists, so while more than 90% of plastic surgeons considered the prophylactic excision to be the best course of action, only a third of dermatologists had the same opinion.^26^ Moreover, plastic surgeons more commonly recommended that the excision be performed in childhood, in contrast to dermatologists, who chose to wait until adulthood.^26^

In addition to the excision, other methods are frequently used to treat and improve Jadassohn lesions, such as curettage, cauterization, cryotherapy, photodynamic therapy,^15^, ^25^, ^27^ topical salicylic acid, topical and systemic retinoids, topical application of vitamin D analog, laser treatment, and dermabrasion.^3^, ^15^, ^25^

In individuals with greater system impairment due to Schimmelpenning-Feuerstein-Mims syndrome, a multidisciplinary approach to treatment is recommended, with the collaboration of a dermatologist, pediatrician, neurologist, ophthalmologist, geneticist,^7^ or any other subspecialist, if necessary.^2^, ^7^ The use of dermoscopy to monitor possible complications is also indicated.^16^, ^28^

## Conclusion

Although the Schimmelpenning-Feuerstein-Mims syndrome constitutes a rare manifestation, it is important to note that its diagnosis should be considered in children born with nevus sebaceous, when it is also associated with abnormalities at the systemic level, thus requiring correct evaluation and management, aiming at minimizing its extracutaneous complications. Moreover, even though there is no consensus on the best therapeutic approach for nevus sebaceous, surgical removal is often reported as a viable alternative, considering the aesthetic aspect and patient well-being regarding self-esteem, in addition to the risk of lesion malignancy, even though it is extremely low.

## Financial support

This research did not receive any specific funding from public, private or non-profit funding agencies.

## Authors’ contributions

Manoel Pereira da Silva Neto: Approval of the final version of the manuscript; design and planning of the study; effective participation in research orientation; drafting and editing of the manuscript; critical review of the literature; critical review of the manuscript.

Barbara Rodovalho de Assis: Approval of the final version of the manuscript; drafting and editing of the manuscript; critical review of the literature; critical review of the manuscript.

Gustavo Rodrigues Andrade: Approval of the final version of the manuscript; drafting and editing of the manuscript; critical review of the literature; critical review of the manuscript.

## Conflicts of interest

The authors have no conflict of interest in this article.
